# Molecular Epidemiology of Ticks and Tick-Borne Pathogens in the Ta-Pa Mountain Area of Chongqing, China

**DOI:** 10.3390/pathogens13110948

**Published:** 2024-10-31

**Authors:** Lijun Wang, Zhongqiu Teng, Li Wan, Wen Wang, Shan Yuan, Qingzhu Huang, Juan Huang, Na Zhao, Meijia Wang, Kun Cao, Hai Huang, Jianguo Xu, Yi Yuan, Tian Qin

**Affiliations:** 1 Chengkou County Center for Disease Control and Prevention, Chongqing 405900, China; wanglijunmail96@163.com (L.W.); 17726693919@163.com (L.W.); 13896219563@163.com (S.Y.); 18996616604@163.com (J.H.); 17783512616@163.com (M.W.); s59905131@163.com (K.C.); 19115508269@163.com (H.H.); 2 National Key Laboratory of Intelligent Tracking and Forecasting for Infectious Diseases, National Institute for Communicable Disease Control and Prevention, Chinese Center for Disease Control and Prevention, Beijing 102206, China; tengzhongqiu@icdc.cn (Z.T.); wangwen@icdc.cn (W.W.); hqz13666941794@126.com (Q.H.); zhaona@icdc.cn (N.Z.); xujianguo@icdc.cn (J.X.)

**Keywords:** tick, *Rickettsia*, *Anaplasma*, *Ehrlichia*, *Coxiella burnetii*, *Babesia*, *Theileria*

## Abstract

To validate the prevalence and biodiversity of ticks and tick-borne pathogens in Chongqing, a total of 601 ticks were collected from dogs, cattle, and goats within the Ta-pa Mountain range in Chongqing, China. Five distinct tick species were identified, including *Ixodes ovatus* (1.66%, 10/601), *I. acutitarsus* (0.50%, 3/601), *Haemaphysalis flava* (10.32%, 62/601), *Ha. hystricis* (9.82%, 59/601), and *Ha. longicornis* (77.70%, 467/601). A suit of semi-nest PCR and nest PCR primers were custom-synthesized for the detection of tick-borne pathogens. The analysis yielded positive results for 7.15% *Rickettsia* (*Candidatus* R. principis, *R. japonica*, and *R. raoultii*), 3.49% *Anaplasma* (*A. bovis* and *A. capra*), 1.16% *Ehrlichia*, 1.83% *Coxiella burnetii*, and 3.49% protozoa (*Theileria. capreoli*, *T. orientalis*, *T. luwenshuni*, and *Babesia* sp.) in ticks. Notably, Ca. R. principis was identified for the first time in *I. ovatus* and *Ha. longicornis*. These findings underscore the significant prevalence and diversity of ticks and their associated pathogens within the Chongqing Ta-pa Mountain region. This study accordingly provides an extensive dataset that contributes to the epidemiological understanding and disease prevention strategies for tick-borne illnesses in the local area.

## 1. Introduction

Ticks are obligate hematophagous ectoparasites of the suborder *Ixodida* that feed on the blood of mammals, birds, reptiles, and other hosts [1,2]. Since Smith and Kilbourne’s discovery, in the late 19th century, that ticks transmit babesiosis to cattle, ticks have been identified as vectors and reservoirs of pathogens [3,4]. Even today, ticks are the most prominent vectors of disease-causing pathogens in domestic and wild animals, second only to mosquitoes worldwide as vectors of human diseases [2]. A diverse array of tick-borne pathogens has been documented globally, encompassing bacteria, protozoa, helminths, and viruses. In addition, an explosive increase in the population of ticks and an expanding range of their activity have been observed, leading to the proliferation of suitable habitats for these arthropod vectors and the pathogens they transmit [5].

Tick-borne diseases (TBDs) have emerged as a significant global public health concern [6]. Tick-borne diseases such as rickettsioses, ehrlichiosis, Lyme disease, Q fever, and protozoan parasites are prevalent worldwide. For instance, between 1906 and 2021, a total of 66,133 human cases of spotted fever group of rickettsiae (SFGR) infections were reported worldwide, especially in North America, the Mediterranean region, and East Asia [7]. Among all 48 SFGR species, 46 species are found in 140 species of hard ticks that belong to seven genera; of these, 24 are known to be associated with human infections. The prevalence of human granulocytic anaplasmosis (HGA) caused by *A. phagocytophilum* has greatly increased in the USA (351 cases in 2000, 1053 cases in 2008, and 3656 cases in 2015), and sporadic and clustered cases have been reported in Europe and China [8,9]. Lyme disease is highly prevalent in moderate climates of the northern hemisphere, and it is estimated that approximately 476,000 cases are diagnosed and treated annually in the United States, and over 200,000 cases per year in Europe [10]. Q fever infections have occurred in numerous countries, including Spain, Switzerland, Great Britain, Germany, France, the United States, Australia, and China [11]. Unprecedented outbreaks of the Q fever epidemic were reported in the Netherlands from 2007 to 2010, with over 4000 identified human cases and 74 deaths [12,13]. Furthermore, about 80% of the world’s cattle population is affected by ticks and tick-borne pathogens, which causes severe economic losses due to the costs associated with parasite control, as well as due to reduced fertility, body weight, and milk production [14].

In China, as the incidence of TBDs has risen in recent years, there has been a heightened focus on, as well as extensive research conducted into, ticks and their vector-borne agents. Approximately 124 tick species and over 100 tick-borne agents have been documented, with records spanning 1134 counties, representing roughly 39% of all counties on the Chinese mainland [15]. By the end of 2018, a total of 2786, 415, 215, 129, and 95 human cases had been confirmed for infections with *Borrelia*, *Anaplasmataceae*, *Babesia* spp., SFGR, and *Co. burnetii* in China, respectively [15]. The majority of tick bites occur unbeknownst to the affected individual, and they can present with atypical or chronic symptoms, which can be diagnostically challenging to differentiate from one another. This may pose a higher risk of morbidity or mortality for older adults, individuals with underlying health conditions, or those with weakened immune systems [5]. Consequently, a comprehensive understanding of ticks and tick-borne pathogens in various regions is crucial for more effective prevention and management of locally acquired TBDs.

Chongqing (28°10′~32°13′ N, 105°11′~110°11′ E) is located in the southwestern region of China. The municipality features a subtropical monsoon humid climate, characterized by high humidity, and a relatively low annual sunshine duration. Reports of human infections with Lyme disease and babesiosis caused by the species *Bo. afzelii* and uncharacterized species of *Babesia*, respectively, within the region of Chongqing highlight the possible risks associated with tick-borne pathogens [16,17]. In recent years, the expansion of tea cultivation, livestock breeding, and agricultural operations may serve to heighten the risk of infection among both humans and domestic animals. Nevertheless, the 2019 National Tick Monitoring Report of China has revealed a significant knowledge gap in ticks and their associated pathogens due to the lack of effective sampling strategies and robust surveillance mechanisms [18]. To determine the prevalence and biodiversity of ticks and vector-borne agents, and to devise an effective sampling strategy for subsequent surveillance efforts within the region, our team conducted tick surveillance in Chengkou County, Chongqing.

## 2. Materials and Methods

### 2.1. Specimen Collection and Identification

**Figure 1:**
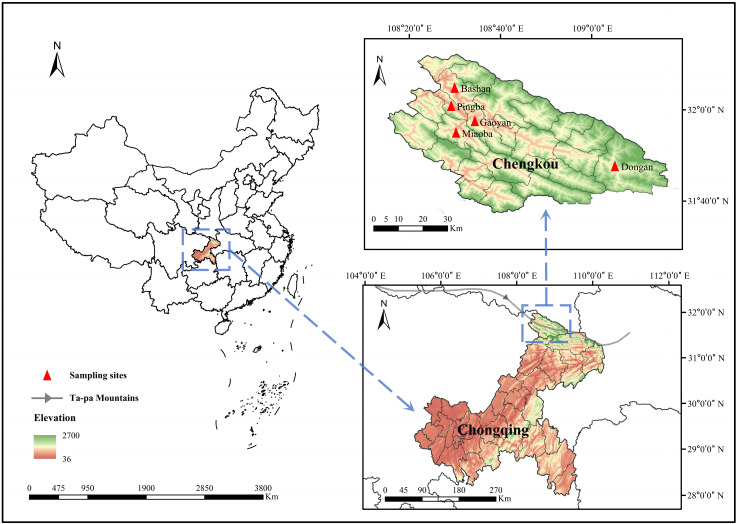
Sampling sites in Chengkou County, Chongqing.

Chengkou County (31°37′~32°13′ N, 108°15′~109°16′ E), a jurisdiction within the municipality of Chongqing, boasts a north subtropical mountainous climate. It is located at the southern base of the Ta-pa Mountains (Figure 1). Between May and July 2024 (average temperature at 19.5–25 °C, relative humidity at 74–82%), a total of 601 questing ticks were collected from the surfaces of goats, cattle, and dogs in Chengkou, while no free-living ticks were harvested by the drag–flag method within the environment. Ticks were identified using a light microscope referring to the standard taxonomic keys, followed by polymerase chain reaction (PCR) amplification based on the mitochondrial COI gene [19]. The primers targeting the 16S rRNA gene sequence were employed for the subsequent identification of tick species [20]. Primers for ticks and tick-borne pathogens detection are depicted in Table S1.

### 2.2. Nucleic Acid Extraction

Ticks were washed with bromogeramine (5%), alcohol (75%), and phosphate-buffered saline (PBS) individually for 15 min each. Following air-drying, the ticks were individually homogenized, and DNA extraction was performed using the QIAamp DNA Mini Kit (Qiagen, Hilden, Germany). All DNA samples were stored at −20 °C.

### 2.3. PCR Assays and Sequencing

PCR, nested PCR, or semi-nested PCR amplification was employed to detect genes associated with ticks and tick-borne pathogens, including *Rickettsia* spp. [21], *Anaplasma* spp. [22,23,24,25], *Ehrlichia* spp. [1], *Coxiella* spp. [26], *Borrelia* spp. [27,28], *Babesia* spp., *Theileria* spp., and *Hepatozoon* spp. [29] (Table S1). Primers were custom-synthesized by Sangon Biotech Co., Ltd. (Shanghai, China). Fragments of the anticipated size were verified through agarose gel electrophoresis and subsequent Sanger sequencing (Tianyi Huiyuan Biotechnology Company, Beijing, China).

### 2.4. Phylogenetic Analyses

Sequences were edited and assembled using the SeqMan software (DNASTAR, Madison, WI, USA, SeqMan Pro 12.1.0). Basic Local Alignment Search Tool (BLAST) analyses were conducted to compare them with sequences available in GenBank. Furthermore, the neighbor-joining (NJ) method was employed for multiple alignments, resulting in the construction of a phylogenetic tree in MEGA 7.0. To evaluate the reliability of the results, a bootstrap method with 1000 replications was utilized. All representative sequences were deposited in GenBank.

## 3. Results

### 3.1. Tick Species Identification

**Figure 2:**
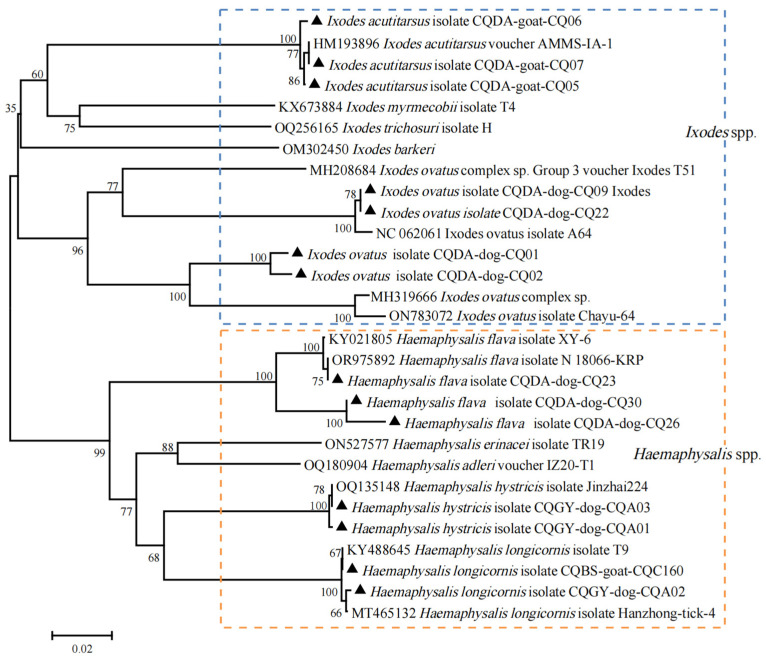
Phylogenetic analysis of ticks based on the nucleotide sequences of *COI*. Sequences obtained in this study are marked with black triangles before their names.

A total of 601 adult ticks were collected from Dongan, Miaoba, Pingba, Gaoyan, and Bashan, five towns in Chengkou in the Ta-pa Mountain area (Figure 1, Table S2). And a map was constructed based on the Digital Mountain Map of China dataset [30,31]. According to the results of the morphological examination and COI sequence analysis, five specific tick species were identified as *I. ovatus* (1.66%, 10/601), *I. acutitarsus* (0.50%, 3/601), *Ha. flava* (10.32%, 62/601), *Ha. hystricis* (9.82%, 59/601), and *Ha. longicornis* (77.70%, 467/601). In the *I. ovatus* group, eight ticks showed 90.02–90.79% identity (Query cover: 100%, E-value: 0.0) with previously reported *I. ovatus* (MH319666), while they were still on the same branch in the phylogenetic tree. Further molecular analysis utilizing the 16S rRNA gene sequence revealed that these ticks exhibited a sequence similarity of 96.15–96.65% (Query cover: 100%, E-value ≤ 1 × 10^−88^) when matched against sequences documented from the China–Myanmar border county (MH319616, MH319598). They were also positioned within the *I. ovatus* clade in the phylogenetic tree (Table S3, Figure S1). Additionally, five ticks of the *Ha. flava* species showed 97.51–94.52% identity (Query cover: 100%, E-value: 0.0) with those found in other regions of China (KY021805, Figure 2). Other COI gene sequences of ticks shared 99.00–100.00% identity with those of the aforementioned five tick species present in GenBank (Table S3). In Chengkou County, *Ha. longicornis* exhibits the highest prevalence, with specimens being recovered from Miaoba, Gaoyan, and Bashan. The main hosts include cattle and goats, along with a few dogs. Specifically, three species (*I. ovatus*, *Ha. flava*, and *Ha. hystricis*) were exclusively found on dogs, while *I. acutitarsus* was solely detected on goats (Table S2).

### 3.2. Tick-Borne Pathogens: Identification and Prevalence Analysis

#### 3.2.1. *Rickettsia* (Total Prevalence 7.15%)

**Figure 3:**
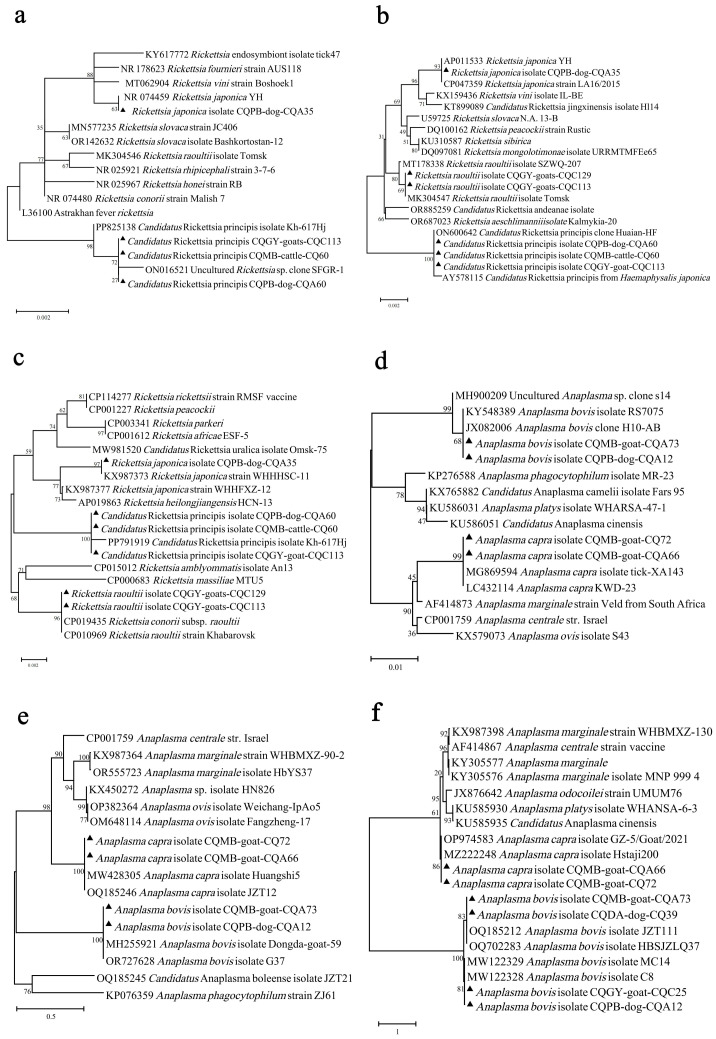
Phylogenetic analysis based on the nucleotide sequences of 16S rRNA (**a**), *glt*A (**b**), and *groEL* (**c**) of *Rickettsia* strains and on the 16S rRNA (**d**), *glt*A (**e**), and *groEL* (**f**) of *Anaplasma* strains. Sequences obtained in this study are marked with black triangles before their names.

Nucleotide alignment and phylogenetic analysis of *Rickettsia* spp. were performed based on the 16S rRNA, *glt*A, and *groEL* genes. In the phylogenetic tree, these genes clustered together with the corresponding genes of Ca. R. principis, *R. japonica*, and *R. raoultii* (negative in 16S rRNA semi-nested PCR), accompanied by high homology (Figure 3a–c). Although one group of *Rickettsia* species was closest to an uncultured *Rickettsia* sp. (ON016521), with 99.92% identity according to the 16S rRNA BLASTN, they also showed high homology (99.91%, Query cover: 81%, E-value: 0.0) with the 16S rRNA of Ca. R. principis (PP825138). Phylogenetic analysis based on the *glt*A and *groEL* genes confirmed these strains as Ca. R. principis. So, we proposed that three *Rickettsia* species were detected in ticks, including Ca. R. principis (6.66%), *R. japonica* (0.17%), and *R. raoultii* (0.33%). Ca. Rickettsia principis existed in *I. ovatus*, *Ha. flava*, and *Ha. Longicornis*, while *R. japonica* and *R. raoultii* were found in *Ha. hystricis* and *Ha. Longicornis*, respectively (Table 1).

#### 3.2.2. *Anaplasma* (Total Prevalence 3.49%)

Analysis based on the 16S rRNA, *glt*A, and *groEL* genes indicated that two *Anaplasma* species were identified in the current study. The phylogenetic tree showed that our sequences were located in the *A. bovis* and *A. capra* cluster (Figure 3d–f). Except for one genotype of *groEL* in *A. bovis*, which was 99.86% similar to the *A. bovis* found in South Korea, others were identical to previous *A. bovis* and *A. capra* (Table S3). In the prevalence analysis, *A. bovis* was detected in 12 (2.00%) ticks, including *Ha. flava* and *Ha. longicornis*. *A. capra* was present in nine (1.50%) ticks, only found in *Ha. longicornis*.

#### 3.2.3. *Ehrlichia* (Total Prevalence 1.16%)

**Figure 4:**
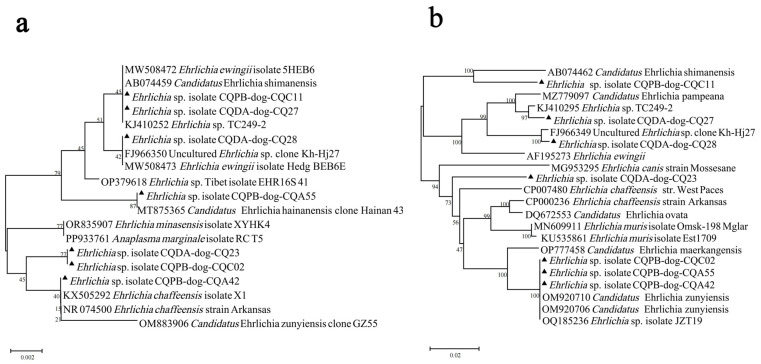
Phylogenetic analysis of *Ehrlichia* strains based on the nucleotide sequences of 16S rRNA (**a**) and *groEL* (**b**). Sequences obtained in this study are marked with black triangles before their names.

*Ehrlichia* sp. was detected in seven (1.16%) ticks based on the genetic sequences of 16S rRNA and *groEL*. The 16S rRNA and *groEL* gene sequences of the strains (CQPB-dog-CQC11, CQDA-dog-CQ27, CQPB-dog-CQA55, CQPB-dog-CQC02, and CQPB-dog-CQA42) showed 99.12–100.00% identity to diverse *Ehrlichia* species in GenBank. These strains were identified as unspecific *Ehrlichia* sp. Strain CQDA-dog-CQ23 showed the highest identity, at 99.53% (Query cover: 100%, E-value: 0.0) and 94.42% (Query cover: 100%, E-value: 0.0), to the 16S rRNA and *groEL* genes of *E. chaffeensis* str. Arkansas, respectively. Another strain, CQDA-dog-CQ28, showed the highest identity, at 100.00% (Query cover: 100%, E-value: 0.0) and 94.41% (Query cover: 100%, E-value: 0.0), to the 16S rRNA and *groEL* genes of uncultured *Ehrlichia* sp. clone Kh-Hj27 from Russia, respectively (Figure 4, Table S3).

#### 3.2.4. *Coxiella burnetii* (Total Prevalence 1.83%)

**Figure 5:**
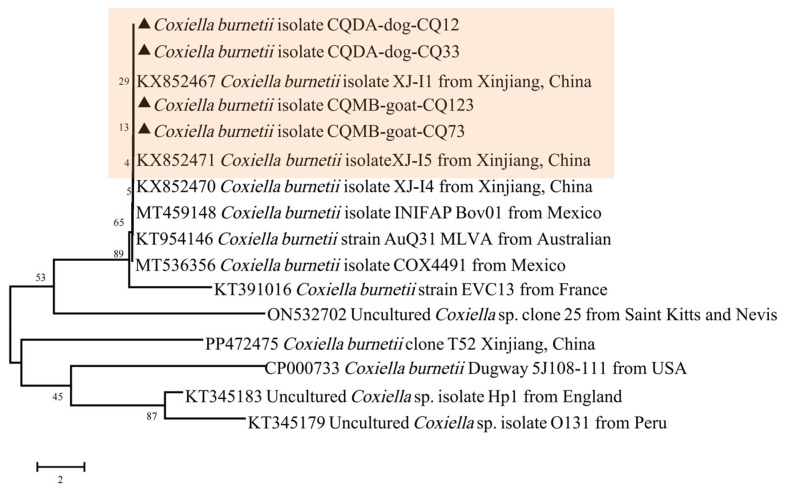
Phylogenetic analysis of *Co. burnetii* strains based on the nucleotide sequences of IS*1111*. Sequences obtained in this study are marked with black triangles before their names.

*I. ovatus*, *Ha. flava*, and *Ha. longicornis* were confirmed to harbor *Co. burnetii, with* positive strains detected in eight (1.83%) ticks from Dongan and Miaoba. These IS*1111*-positive sequences were identical to those obtained from the previous strains and clustered together with *Co. burnetii*, as reported in Xinjiang, in a phylogenetic tree (Figure 5).

#### 3.2.5. Protozoa (Total Prevalence 3.49%)

**Figure 6:**
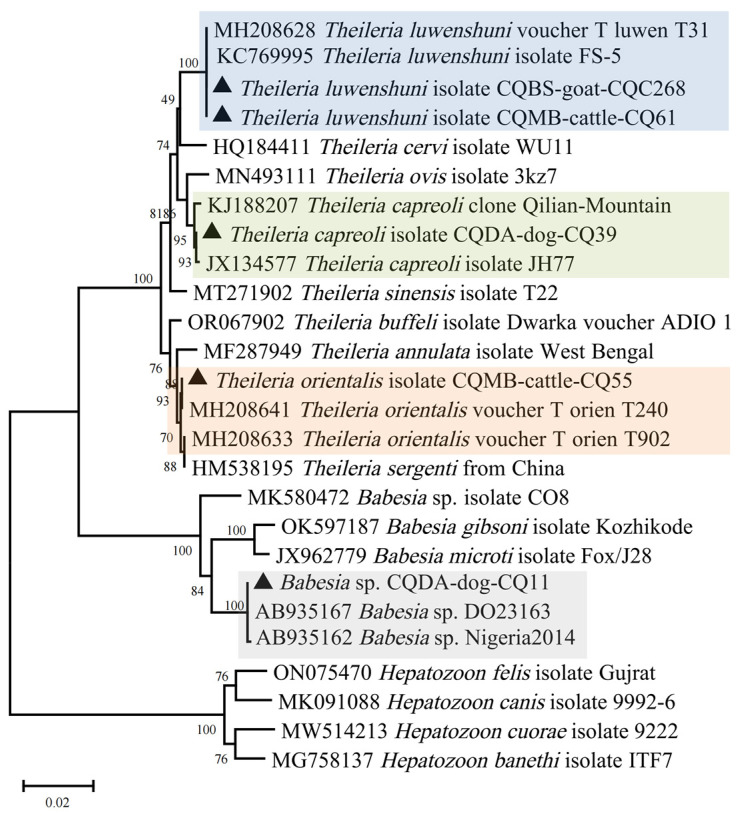
Phylogenetic analysis of *Theileria* and *Babesia* based on the nucleotide sequences of 18S rRNA. Sequences obtained in this study are marked with black triangles before their names.

Primers based on the 18S rRNA gene for *Babesia* spp., *Theileria* spp., and *Hepatozoon* spp. were used in this study [29]. *Babesia* sp. was detected in one *Ha. flava* tick (0.17%) which was sorted in the same cluster as *Babesia* sp. from Japan (AB935167) (Figure 6), with identical 18S rRNA gene sequences. In addition, three *Theileria* species were detected in Chongqing, including *T. capreoli* (0.17%), *T. orientalis* (0.17%), and *T. luwenshuni* (3.00%). *T. luwenshuni*, detected in ticks from goats and cattle, was the dominant protozoan in Dongan, Miaoba, Gaoyan, and Bashan. A *Ha. flava* collected from a dog in Dongan was found to be infected with *T. capreoli*. Its 18S rRNA gene showed 99.93% identity with that detected in the blood of a white-lipped deer (JX134577). *T. orientalis* was carried by a *Ha. longicornis* collected from cattle in Miaoba, with an identical 18S rRNA gene sequence to that of *T. orientalis* isolated in *Rhipicephalus microplus* (MH208641).

#### 3.2.6. *Borrelia*

No strains were detected using nested PCR based on the *osp*A and 16S rRNA genes of *Borrelia* spp. in this study.

## 4. Discussion

A total of 601 hard ticks collected from Chengkou County within the Ta-pa Mountains were identified. The species consisted of *I. ovatus*, *I. acutitarsus*, *Ha. flava*, *Ha. hystricis*, and *Ha. longicornis*. Furthermore, eight individuals of the species *I. ovatus* showed 90.02–90.79% identity with those collected in a China–Myanmar border county, and five samples of the species *Ha. flava* demonstrated an identity of 97.51% to 94.52% with counterparts distributed across various regions of China. Our findings underscore the biodiversity of tick populations within the Chongqing area. In terms of prevalence, *Ha. longicornis* is the predominant hard tick species (77.70%, 467/601) in Chengkou, hosting various pathogens, including Ca. R. principis, *R. raoultii*, *A. capra*, *A. bovis*, *T. orientalis*, *T. luwenshuni*, and *Co. burnetii*. This species is also the predominant tick in China and has been reported in at least 17 provinces and associated with over 49 pathogen species, including pathogens that are responsible for confirmed human infections like *Rickettsia*, *Anaplasmataceae*, *Borrelia*, *Babesia* spp., various viruses, and so on [15,32]. This highlights the potential risk of tick-borne pathogens of *Ha. longicornis* in Chongqing. And there is an immediate necessity to intensify the control and surveillance of local tick populations. In terms of the overall prevalence of positive pathogen detection rates, the tick species *Ha. flava* represents the most significant threat, with 64.91% (37/57), whereas *Ha. longicornis* was found in 8.75% of the individuals surveyed and found to be infected with pathogens, including Ca. R. principis, *A. bovis*, *Ehrlichia* sp., *Babesia* sp., *T. capreoli*, and *Co. burnetii*. Reports have revealed a broad distribution of *Ha. flava* across Asia, encompassing regions such as China, Japan, Vietnam, and South Korea [33]. The species is capable of inflicting bites on a diverse array of hosts, which include humans, domestic animals, and various wildlife [34,35,36]. All *Ha. flava* individuals in the present study were sourced from canine hosts, highlighting the critical role that dogs play in the transmission and reservoir maintenance of ticks. Strategies for local tick and tick-borne disease prevention and control should prioritize pet owners’ implementation of appropriate deworming and husbandry practices for their dogs.

Among the tick-borne bacteria identified in the Chongqing Ta-pa Mountain area, *Rickettsia* species exhibited the highest overall prevalence rate, accounting for 7.15% of the ticks. This finding suggests that heightened attention should be given to the prevention of rickettsioses. Three *Rickettsia* species (Ca. R. principis, *R. japonica*, and *R. raoultii*) were identified in reference to the criteria for identifying novel *Rickettsia* sp. based on gene sequences established previously [37]. Ca. R. principis was the predominant species among these three *Rickettsia* species, and it also represented the dominant species among all tick-borne pathogens in this study. Notably, it displayed a higher prevalence in *Ha. flava* (57.89%, 33/57) than in ticks of the species *Ixodes* (15.38%, 2/13) or *Ha. longicornis* (0.43%, 2/467). To the best of our knowledge, Ca. R. principis was detected for the first time in *I. ovatus* and *Ha. Longicornis*, and this strain had been found in *Ha. japonica*, *Ha. megaspinosa*, *Ha. flava*, *Ha. danieli*, *Ha. qinghaiensis*, and *I. persulcatus* ticks according to previous reports [38,39,40,41,42]. Although the pathogenicity of this *Rickettsia* species to humans remains unknown, further investigation and risk mitigation measures are warranted, given that many *Rickettsia* species previously considered non-pathogenic are now associated with human diseases [43]. Additionally, this study identified two other *Rickettsia* species, *R. japonica* and *R. raoultii*, as being responsible for infectious diseases in humans [42,44,45]. The data suggest a risk of rickettsioses in Chongqing. It is crucial that healthcare personnel in the local area be equipped with training in the identification, diagnosis, and management of these conditions.

In addition to the aforementioned findings, the presence of other tick-borne pathogens capable of causing severe zoonotic diseases has been confirmed in Chongqing. Anaplasmosis can be caused by *A. bovis* and *A. capra* [8,46], and these two *Anaplasma* species were detected at an overall prevalence rate of 3.49% in the Chongqing Ta-pa Mountain area. We suggest that local farmers need to enhance preventive and control measures during the summer season, as infection rates are markedly higher then compared to other seasons [47]. *Co. burnetii* serves as the etiological agent of Q fever, and its presence has been identified in Chongqing. Farmers and agricultural workers should receive comprehensive training to guard themselves against infections, as individuals are particularly susceptible to infection through the inhalation of contaminated air or direct contact with animals harboring the pathogen and their secretions, such as excreta, urine, milk, and other bodily fluids [48,49]. The detection of *Ehrlichia* species in Chongqing underscores the peril of ehrlichioses. The presence of numerous *Ehrlichia* genotypes in Chongqing denotes the intricacy of these strains, indicating that they remain to be fully elucidated.

In the screening of protozoa, three *Theileria* species (*T. capreoli*, *T. orientalis*, and *T. luwenshuni*) and a *Babesia* species were identified. *Theileria* species are known to infect both domestic animals and wildlife, and *Ha. longicornis* is particularly recognized as a key transmission vector for these parasites [50,51,52]. The prevalence of *Ha. longicornis* in Chongqing indicates a potential risk of theileriosis. To alleviate the economic losses sustained by grazers and to safeguard the wildlife population within the Chongqing Ta-pa Mountain area, it is recommended that livestock farmers in these areas (particularly those who raise free-range livestock) adopt preventive measures to forestall the cross-contamination of *Theileria* species between domestic livestock and wildlife via tick vectors. It is noteworthy that the detection of *Babesia* species in Chengkou County serves as a reminder of the series of human parasitemia incidents attributed to suspected *Babesia* species that occurred from 1931 to 1944 in Beibei, Chongqing [17]. Regrettably, due to age, we cannot obtain more relevant data to prove whether the *Babesia* sp. we obtained are related to the parasites reported in the literature. This also underscores the necessity for stringent preventive and control measures to forestall the re-emergence of diseases caused by *Babesia* species.

Overall, the absence of reports involving tick bite cases in Chongqing has led to a persistent underestimation of the risk associated with tick-borne diseases in that region. Our study has demonstrated the biodiversity and prevalence of ticks and tick-borne pathogens in Chongqing, offering an effective sampling strategy for subsequent surveillance efforts within the region. Concurrently, we have provided appropriate prevention and control recommendations for the risk points posed by ticks and tick-borne pathogens in the local area. However, limitations do exist within this study. The acquisition of ticks was limited to a few livestock species, including goats, cattle, and dogs, and the drag–flag method did not acquire any free-living tick samples. This limitation has narrowed the ecological scope of this study, which could potentially impact the assessment of tick biodiversity and the incidence of associated pathogens. In 2011, *B. afzelii* strains were isolated from the blood of patients in Chongqing [16]. The authors emphasize that *Ha. bispinosa* could potentially act as one of the vectors for Lyme disease transmission in southern China. The absence of the detection of *Ha. bispinosa* and *Borrelia* spp. during the course of our investigation highlights the substantial geographical variation exhibited by these tick species and the associated pathogens they harbor. Furthermore, this lacuna underscores the inherent limitations of our current study, thereby underscoring the imperative need for enhanced and continuous surveillance initiatives within the Chongqing area.

## 5. Conclusions

This study represents the first comprehensive documentation of the prevalence and biodiversity of ticks and tick-borne pathogens in the Chongqing Ta-pa Mountain area. Five distinct tick species, including *I. ovatus*, *I. acutitarsus*, *Ha. flava*, *Ha. hystricis*, and *Ha. longicornis*, have been identified. The positive detection of *Rickettsia* (Ca. R. principis, *R. japonica*, and *R. raoultii*), *Anaplasma* (*A. bovis* and *A. capra*), *Ehrlichia* sp., *Co. burnetii*, and protozoa (*T. capreoli*, *T. orientalis*, *T. luwenshuni*, and *Babesia* sp.) suggests that public health officials need to be vigilant about potential risks. This report has yielded some recommendations for these possible risks, and it also affords a viable sampling strategy that can be adopted by local communities. This would be helpful in the development and execution of regional strategies aimed at the prevention and control of tick-borne diseases. Nevertheless, the temporal, geographical, and source restrictions of sampling, which were exclusively obtained from goats, cattle, and dogs, impose limitations on our dataset. Consequently, there is a pressing need for subsequent continuous and comprehensive surveillance efforts.

## Figures and Tables

**Table 1:** Prevalence of tick-borne pathogens in 601 ticks collected from the Ta-pa Mountain area in Chongqing, China.

Pathogen Species	Tick Species (Positive Ticks No.)	Host (Positive Ticks No.)	Geographical Distribution (Positive Ticks No.)	Total Prevalence %
Ca. R. principis	*Ha. flava* (36), *Ha. longicornis* (2), *I. ovatus* (2)	dogs (38), goats (1), cattle (1)	Dongan (24), Miaoba (1), Pingba (14), Gaoyan (1)	6.66% (40/601)
*R. japonica*	*Ha. hystricis* (1)	dogs (1)	Pingba (1)	0.17% (1/601)
*R. raoultii*	*Ha. longicornis* (2)	goats (2)	Gaoyan (2)	0.33% (2/601)
*Ehrlichia* sp.	*Ha. flava* (4), *Ha. hystricis* (3)	dogs (7)	Dongan (3), Pingba (4)	1.16% (7/601)
*A. bovis*	*Ha. flava* (2), *Ha. longicornis* (10)	dogs (2), goats (10)	Dongan (1), Miaoba (5), Pingba (1), Gaoyan (3), Bashan (2)	2.00% (12/601)
*A. capra*	*Ha. longicornis* (9)	goats (9)	Miaoba (9)	1.50% (9/601)
*Babesia* sp.	*Ha. flava* (1)	dogs (1)	Dongan (1)	0.17% (1/601)
*T. capreoli*	*Ha. flava* (1)	dogs (1)	Dongan (1)	0.17% (1/601)
*T. orientalis*	*Ha. longicornis* (1)	cattle (1)	Miaoba (1)	0.17% (1/601)
*T. luwenshuni*	*I. acutitarsus* (1), *Ha. longicornis* (17)	goats (17), cattle (1)	Dongan (1), Miaoba (14), Gaoyan (2), Bashan (1)	3.00% (18/601)
*Co. burnetii*	*I. ovatus* (3), *Ha. flava* (3), *Ha. longicornis* (5)	dogs (6), goats (4), cattle (1)	Dongan (6), Miaoba (5)	1.83% (11/601)

## Data Availability

All relevant data are within the paper and its Supplementary Materials files.

## Supplementary Materials

The following supporting information can be downloaded at https://www.mdpi.com/article/10.3390/pathogens13110948/s1, Figure S1: Phylogenetic analysis of ticks based on the nucleotide sequences of 16S rRNA.; Table S1: Primers for ticks and tick-borne pathogen detection. Table S2: Tick prevalence in Chengkou, Chongqing, China. Table S3: List of nucleotide sequence accession numbers and identities with BLAST.

## References

[1] Teng Z., Shi Y., Zhao N., Zhang X., Jin X., He J., Xu B., Qin T. (2023). Molecular Detection of Tick-Borne Bacterial and Protozoan Pathogens in *Haemaphysalis longicornis* (Acari: Ixodidae) Ticks from Free-Ranging Domestic Sheep in Hebei Province, China. Pathogens.

[2] Fuente J.d.l. (2008). Overview: Ticks as vectors of pathogens that cause disease in humans and animals. Front. Biosci..

[3] Bowman A.S., Nuttall P.A., Chappell L.H. (2005). Ticks: Biology, disease and control. Parasitology.

[4] Parola P., Raoult D. (2000). Ticks and tickborne bacterial diseases in humans: An emerging infectious threat. Clin. Infect. Dis..

[5] Madison-Antenucci S., Kramer L.D., Gebhardt L.L., Kauffman E. (2020). Emerging Tick-Borne Diseases. Clin. Microbiol. Rev..

[6] Michelet L., Delannoy S., Devillers E., Umhang G.R., Aspan A., Juremalm M., Chirico J., van der Wal F.J., Sprong H., Boye Pihl T.P. (2014). High-throughput screening of tick-borne pathogens in Europe. Front. Cell. Infect. Microbiol..

[7] Zhang Y.-Y., Sun Y.-Q., Chen J.-J., Teng A.-Y., Wang T., Li H., Hay S.I., Fang L.-Q., Yang Y., Liu W. (2023). Mapping the global distribution of spotted fever group rickettsiae: A systematic review with modelling analysis. Lancet Digit. Health.

[8] Li H., Zheng Y.-C., Ma L., Jia N., Jiang B.-G., Jiang R.-R., Huo Q.-B., Wang Y.-W., Liu H.-B., Chu Y.-L. (2015). Human infection with a novel tick-borne *Anaplasma* species in China: A surveillance study. Lancet Infect. Dis..

[9] Adams D.A., Thomas K.R., Jajosky R.A., Foster L., Baroi G. (2017). Summary of Notifiable Infectious Diseasesand Conditions—United States, 2015. MMWR Morb. Mortal. Wkly. Rep..

[10] Koutantou M., Drancourt M., Angelakis E. (2024). Prevalence of Lyme Disease and Relapsing Fever *Borrelia* spp. in Vectors, Animals, and Humans within a One Health Approach in Mediterranean Countries. Pathogens.

[11] Huang M., Ma J., Jiao J., Li C., Chen L., Zhu Z., Ruan F., Xing L., Zheng X., Fu M. (2021). The epidemic of Q fever in 2018 to 2019 in Zhuhai city of China determined by metagenomic next-generation sequencing. PLoS Negl. Trop. Dis..

[12] Roest H.I.J., Tilburg J.J.H.C., Van Der Hoek W., Vellema P., Van Zijderveld F.G., Klaassen C.H.W., Raoult D. (2010). The Q fever epidemic in The Netherlands: History, onset, response and reflection. Epidemiol. Infect..

[13] Schimmer B., Dijkstra F., Vellema P., Schneeberger P.M., Hackert V., ter Schegget R., Wijkmans C., van Duynhoven Y., van der Hoek W. (2009). Sustained intensive transmission of Q fever in the south of the Netherlands, 2009. Eurosurveillance.

[14] Valente D., Carolino N., Gomes J., Coelho A.C., Espadinha P., Pais J., Carolino I. (2024). A study of knowledge, attitudes, and practices on ticks and tick-borne diseases of cattle among breeders of two bovine Portuguese autochthonous breeds. Vet. Parasitol. Reg. Stud. Rep..

[15] Zhao G.-P., Wang Y.-X., Fan Z.-W., Ji Y., Liu M.-J., Zhang W.-H., Li X.-L., Zhou S.-X., Li H., Liang S. (2021). Mapping ticks and tick-borne pathogens in China. Nat. Commun..

[16] Hao Q., Hou X., Geng Z., Wan K. (2011). Distribution of *Borrelia burgdorferi* Sensu Lato in China. J. Clin. Microbiol..

[17] Zhou X., Xia S., Huang J.-L., Tambo E., Zhuge H.-X., Zhou X.-N. (2014). Human babesiosis, an emerging tick-borne disease in the People’s Republic of China. Parasites Vectors.

[18] Wu H.-X., Liu X.-B., Yue Y.-J., Lu L., Ren D.-S., Wang J., Li G.-G., Zhao N., Song X.-P., Liu Q.-Y. (2020). National surveillance report on ticks in China, 2019. Chin. J. Vector Biol. Control.

[19] Wang J., Yang J., Gao S., Liu A., Rashid M., Li Y., Liu Z., Liu J., Liu G., Luo J. (2020). Rapid detection and differentiation of *Theileria annulata*, *T. orientalis* and *T. sinensis* using high-resolution melting analysis. Ticks Tick-Borne Dis..

[20] Krakowetz C.N., Lindsay L.R., Chilton N.B. (2011). Genetic diversity in Ixodes scapularis (Acari: Ixodidae) from six established populations in Canada. Ticks Tick-Borne Dis..

[21] Guo W.-P., Tian J.-H., Lin X.-D., Ni X.-B., Chen X.-P., Liao Y., Yang S.-Y., Dumler J.S., Holmes E.C., Zhang Y.-Z. (2016). Extensive genetic diversity of Rickettsiales bacteria in multiple mosquito species. Sci. Rep..

[22] Jafar Bekloo A., Ramzgouyan M.R., Shirian S., Faghihi F., Bakhshi H., Naseri F., Sedaghat M., Telmadarraiy Z. (2018). Molecular Characterization and Phylogenetic Analysis of *Anaplasma* spp. and *Ehrlichia* spp. Isolated from Various Ticks in Southeastern and Northwestern Regions of Iran. Vector-Borne Zoonotic Dis..

[23] Guo W.-P., Tie W.-F., Meng S., Li D., Wang J.-L., Du L.-Y., Xie G.-C. (2020). Extensive genetic diversity of *Anaplasma bovis* in ruminants in Xi’an, China. Ticks Tick-Borne Dis..

[24] Coburn J., Guo W.-P., Huang B., Zhao Q., Xu G., Liu B., Wang Y.-H., Zhou E.-M. (2018). Human-pathogenic *Anaplasma* spp., and *Rickettsia* spp. in animals in Xi’an, China. PLoS Negl. Trop. Dis..

[25] Remesar S., Prieto A., García-Dios D., López-Lorenzo G., Martínez-Calabuig N., Díaz-Cao J.M., Panadero R., López C.M., Fernández G., Díez-Baños P. (2021). Diversity of *Anaplasma* species and importance of mixed infections in roe deer from Spain. Transbound. Emerg. Dis..

[26] Fenollar F., Fournier P.E., Raoult D. (2004). Molecular Detection of *Coxiella burnetii* in the Sera of Patients with Q Fever Endocarditis or Vascular Infection. J. Clin. Microbiol..

[27] Wanchun T., Zhikun Z., Moldenhauer S., Yixiu G., Qiuli Y., Lina W., Mei C. (1998). Detection of *Borrelia burgdorferi* from Ticks (Acari) in Hebei Province, China. J. Med. Entomol..

[28] Zhang L., Miao G., Hou X., Li B., Hao Q. (2018). Evaluation of nested PCR and real-time PCR in host surveillance of Lyme disease. Chin. J. Vector Biol. Control.

[29] Kumar B., Maharana B.R., Thakre B., Brahmbhatt N.N., Joseph J.P. (2022). 18S rRNA Gene-Based Piroplasmid PCR: An Assay for Rapid and Precise Molecular Screening of *Theileria* and *Babesia* Species in Animals. Acta Parasitol..

[30] Nan X., Li A., Deng W. (2022). Data Set of “Digital Mountain Map of China” (2015). A Big Earth Data Platform for Three Poles. https://cstr.cn/18406.11.Terre.tpdc.272523.

[31] Zhao W., Li A., Bian J. (2019). Research Center for Digital Mountain and Remote Sensing Application, Institute of Mountain Hazards and Environment. Mt. Res. Dev..

[32] Chen Z., Yang X., Bu F., Yang X., Yang X., Liu J. (2010). Ticks (Acari: Ixodoidea: Argasidae, Ixodidae) of China. Exp. Appl. Acarol..

[33] Sang M.K., Patnaik H.H., Park J.E., Song D.K., Jeong J.Y., Hong C.E., Kim Y.T., Shin H.J., Ziwei L., Hwang H.J. (2023). Transcriptome analysis of *Haemaphysalis flava* female using Illumina HiSeq 4000 sequencing: De novo assembly, functional annotation and discovery of SSR markers. Parasites Vectors.

[34] Cheng W.-Y., Zhao G.-H., Jia Y.-Q., Bian Q.-Q., Du S.-Z., Fang Y.-Q., Qi M.-Z., Yu S.-K. (2013). Characterization of *Haemaphysalis flava* (Acari: Ixodidae) from Qingling subspecies of giant panda (*Ailuropoda melanoleuca qinlingensis*) in Qinling Mountains (Central China) by morphology and molecular markers. PLoS ONE.

[35] Li Z., Cheng T., Xu X., Song L., Liu G. (2017). Genetic variation in mitochondrial genes of the tick *Haemaphysalis flava* collected from wild hedgehogs in China. Exp. Appl. Acarol..

[36] Kim H.C., Han S.H., Chong S.T., Klein T.A., Choi C.-Y., Nam H.-Y., Chae H.-Y., Lee H., Ko S., Kang J.-G. (2011). Ticks Collected from Selected Mammalian Hosts Surveyed in the Republic of Korea During 2008–2009. Korean J. Parasitol..

[37] Fournier P.-E., Dumler J.S., Greub G., Zhang J., Wu Y., Raoult D. (2003). Gene Sequence-Based Criteria for Identification of New *Rickettsia* Isolates and Description of *Rickettsia heilongjiangensis* sp. nov. J. Clin. Microbiol..

[38] Mediannikov O., Sidelnikov Y., Ivanov L., Fournier P.E., Tarasevich I., Raoult D. (2006). Far Eastern Tick-Borne Rickettsiosis: Identification of Two New Cases and Tick Vector. Ann. N. Y. Acad. Sci..

[39] Igolkina Y., Rar V., Vysochina N., Ivanov L., Tikunov A., Pukhovskaya N., Epikhina T., Golovljova I., Tikunova N. (2018). Genetic variability of *Rickettsia* spp. in *Dermacentor* and *Haemaphysalis* ticks from the Russian Far East. Ticks Tick-Borne Dis..

[40] Qi Y., Ai L., Jiao J., Wang J., Wu D., Wang P., Zhang G., Qin Y., Hu C., Lv R. (2022). High prevalence of *Rickettsia* spp. in ticks from wild hedgehogs rather than domestic bovine in Jiangsu province, Eastern China. Front. Cell. Infect. Microbiol..

[41] Chahan B., Jian Z., Jilintai, Miyahara K., Tanabe S., Xuan X., Sato Y., Moritomo T., Nogami S., Mikami T. (2007). Detection of DNA closely related to ‘*Candidatus* Rickettsia principis’ in *Haemaphysalis danieli* recovered from cattle in Xinjiang Uygur Autonomous Region Area, China. Vet. Parasitol..

[42] Okado K., Adjou Moumouni P.F., Lee S.-H., Sivakumar T., Yokoyama N., Fujisaki K., Suzuki H., Xuan X., Umemiya-Shirafuji R. (2021). Molecular detection of *Borrelia burgdorferi* (*sensu lato*) and *Rickettsia* spp. in hard ticks distributed in Tokachi District, eastern Hokkaido, Japan. Curr. Res. Parasitol. Vector-Borne Dis..

[43] Lu M., Li F., Liao Y., Shen J.-J., Xu J.-M., Chen Y.-Z., Li J.-H., Holmes E.C., Zhang Y.-Z. (2019). Epidemiology and Diversity of *Rickettsiales* Bacteria in Humans and Animals in Jiangsu and Jiangxi provinces, China. Sci. Rep..

[44] Switaj K., Chmielewski T., Borkowski P., Tylewska-Wierzbanowska S., Olszynska-Krowicka M. (2012). Spotted fever rickettsiosis caused by *Rickettsia raoultii*—Case report. Przegl. Epidemiol..

[45] Parola P., Rovery C., Rolain J.M., Brouqui P., Davoust B., Raoult D. (2009). *Rickettsia slovaca* and *R. raoultii* in Tick-borne Rickettsioses. Emerg. Infect. Dis..

[46] Ben Said M., Belkahia H., Messadi L. (2018). *Anaplasma* spp. in North Africa: A review on molecular epidemiology, associated risk factors and genetic characteristics. Ticks Tick-Borne Dis..

[47] Wang K., Yan Y., Zhou Y., Zhao S., Jian F., Wang R., Zhang L., Ning C. (2021). Seasonal dynamics of *Anaplasma* spp. in goats in warm-temperate zone of China. Ticks Tick-Borne Dis..

[48] Rodolakis A., Berri M., Héchard C., Caudron C., Souriau A., Bodier C.C., Blanchard B., Camuset P., Devillechaise P., Natorp J.C. (2007). Comparison of *Coxiella burnetii* Shedding in Milk of Dairy Bovine, Caprine, and Ovine Herds. J. Dairy Sci..

[49] Burns R.J.L., Le K.K., Siengsanun-Lamont J., Blacksell S.D. (2023). A review of coxiellosis (Q fever) and brucellosis in goats and humans: Implications for disease control in smallholder farming systems in Southeast Asia. One Health.

[50] Mans B.J., Pienaar R., Latif A.A. (2015). A review of *Theileria* diagnostics and epidemiology. Int. J. Parasitol. Parasites Wildl..

[51] Luo J., Tan Y., Zhao S., Ren Q., Guan G., Luo J., Yin H., Liu G. (2024). Role of Recognition MicroRNAs in *Hemaphysalis longicornis* and *Theileria orientalis* Interactions. Pathogens.

[52] Lakew B.T., Eastwood S., Walkden-Brown S.W. (2023). Epidemiology and Transmission of *Theileria orientalis* in Australasia. Pathogens.

