# Supplementary material for: Molecular Epidemiology of Ticks and Tick-Borne Pathogens in the Ta-Pa Mountain Area of Chongqing, China
Source: Pathogens. 2024 Oct 31;13(11):948. doi: 10.3390/pathogens13110948 (PMC11597067; doi:10.3390/pathogens13110948)
Supplement: Supplementary file 1 [file pathogens-13-00948-s001.zip › pathogens-3263030-supplementary-new.pdf]

Table S1. Primers for ticks and tick-borne pathogens detection.

Organism	Gene name	PCR method	Primer name	Cycle	Primer sequence 5′ to 3′	Approximate amplicon /bp	Reference
Ticks	COI	PCR	TickCOI-F	1	GGTCAACAAATCATAAAGATATTGG	700	[19]
			TickCOI-R	1	TAAACTTCAGGGTGACCAAAAAATCA		
	16S rRNA	PCR	Tick16S-F	1	CCGGTCTGAACTCAGATCAAGT	400	[20]
			Tick16S-R	1	CTGCTCAATGATTTTTTAAATTGCTGTGG		
Rickettsia	16S rRNA	Semi-Nested PCR	Ric-16SF1	1, 2	GTACGGAATAACTTTTAGAAAT	900	[21]
			Ric-16SR1	1	CATGATGACTTGACRTCGT		
			Ric-16SR2	2	CATCTCACGACACGAGCTG		
	*glt*A	Semi-Nested PCR	R-gltA-F1	1	CCGGGYTTTATGTCTACTGC	900	[21]
			R-gltA-F2	2	CTTTATGTCTACTGCKTCTTG		
			R-gltA-R	1, 2	AGCTGTCTWGGTCTGCTGATT		
	groEL	Nested PCR	Ric-ESL-F1	1	CCATTACATGATAGAATTGCAAT	1100	[21]
			Ric-ESL-F2	2	GAATTGCAATAAAGCCTATCG		
			Ric-ESL-R	1, 2	CCATCATTGCTTTTCTTCTATC		
Anaplasmataceae	16S rRNA	Nested PCR	A&E-rrs-F1	1	GAACGAACGCTGGCGGCAAGC	500	[22]
			A&E-rrs-R1	1	AGTA(T/C)CG(A/G)ACCAGATAGCCGC		
			A&E-rrs-F2	2	TGCATAGGAATCTACCTAGTAG		
			A&E-rrs-R2	2	CTAGGAATTCCGCTATCCTCT		
Anaplasma bovis	*glt*A	Semi-Nested PCR	A.b-gltA-F1	1	TACATCWACWGTAAGAATGG	400	[23]
			A.b-gltA-R1	1, 2	CCRGCAGTDCGTCCCAGTGC		
			A.b-gltA-F2	2	ACWGTAAGAATGGTKGGCTC		
	groEL	Semi-Nested PCR	A.b-groEL-F1	1	GTATGCARTTTGATCGYGGAT	800	[23]
			A.b-groEL-F2	2	GAAGTTGGAAGRGAYGGDGT		
			A.b-groEL-R	1, 2	GCCTTWACAGCDGCAACTTG		
Anaplasma capra	*glt*A	Semi-Nested	AC-CS-F1	1	ATGATCCGGGGTTCCTGTC	1000	[24]

		PCR	AC-CS -R	1, 2	TACAATACCGGAGTAAAAGT		
			AC-CS-F2	2	TGCAGGTCTGAGATAACCT		
	groEL	Nested PCR	Ac gro-ELF1	1	GCGAGGCGTTAGACAAGTCCATT	1100	[25]
			Ac gro-ELR1	1	TCCAGAGATGCGAGCGTGTATAG		
			Ac gro-ELF2	2	TGCACTGCTGGTCCAAAGGGGCT		
			Ac gro-ELR2	2	CAACTTCGCTAGAGCCGCCAACC		
Ehrlichia spp.	*groEL*	Semi-Nested PCR	EhgroEL-F1	1	TGGGCTGGYAATGAAATTGA	1100	[1]
			EhgroEL-R1	1, 2	TCAACAGCAGCTCTAGTTG		
			EhgroEL-F2	2	AACATGGCAAATGTAGTTGT		
Coxiella spp.	IS*1111*	Nested PCR	Q-IS1111-F1	1	TACTGGGTGTTGATATTGC	300	[26]
			Q-IS1111-R1	1	CCGTTTCATCCGCGGTG		
			Q-IS1111-F2	2	GTAAAGTGATCTACACGA		
			Q-IS1111-R2	2	TTAACAGCGCTTGAACGT		
Borrelia spp.	*osp*A	Nested PCR	Bo. OspA F1	1	GGGAATAGGTCTAATATTAGCC	400	[27]
			Bo. OspA R1	1	CACTAATTGTTAAAGTGGAAGT		
			Bo. OspA F2	2	GCAAAATGTTAGCAGCCTTGAT		
			Bo. OspA R2	2	CTGTGTATTCAAGTCTGGC		
	Intergene 5S-23S rRNA	Nested PCR	Bo. rRNA F1	1	CGACCTTCTTCGCCTTAAAGC	300	[28]
			Bo. rRNA R1	1	TAAGCTGACTAATACTAATTACCC		
			Bo. rRNA F2	2	TCCTAGGCATTCACCATA		
			Bo. rRNA R2	2	GAGTTCGCGGGAGA		
Babesia–Theileria-Hepatozoon	18S rRNA	nested PCR	BTH18S-F1	1	GTGAAACTGCGAATGGCTCATTAC	1400	[29]
			BTH18S-R1	1	AAGTGATAAGGTTCACAAAACTTCCC		
			BTH18S-F2	2	GGCTCATTACAACAGTTATAGTTTATTTG		
			BTH18S-R2	2	CGGTCCGAATAATTCACCGGAT		

Table S2. Tick prevalence in Chengkou, Chongqing, China.

Tick species	Host (ticks No.)	Geographical distribution (ticks No.)	Total prevalence%
Ixodes acutitarsus	goat (3)	Dongan (3)	0.50% (3/601)
Ixodes ovatus	dog (10)	Dongan (10)	1.66% (10/601)
Haemaphysalis flava	dog (62)	Dongan (39), Pingba (23)	10.32% (62/601)
Haemaphysalis hystricis	dog (59)	Gaoyan (2), Pingba (57)	9.82% (59/601)
Haemaphysalis longicornis	cattle (78), goat (386), dog (3)	Miaoba (256), Gaoyan (141), Bashan (70)	77.70% (467/601)

Table S3. List of nucleotide sequence accession number and identity with BLAST.

**Organism**	**Gene name**	**Sequence in this study**	**Closest BLAST**		**Identity**
		Accession No.	Descriptions (Accession No.)	Source (geo.)	
Tick	COI	PQ219422-PQ219423	*Ixodes ovatus* (NC_062061)	Hog-badger (Wuhan, China)	99.42%
		PQ219424-PQ219431	*Ixodes ovatus* (MH319666)	Tick (Yunnan, China)	90.02%-90.79%
		PQ219432-PQ219434	*Ixodes acutitarsus* (HM193896)	Tick (Zhangmu, Tibet, China)	99.49%-100.00%
		PQ219435-PQ219438	*Haemaphysalis flava* (OR975892)	(Japan)	100.00%
		PQ219439-PQ219443	*Haemaphysalis flava* (KY021805)	(China)	94.52%-97.51%
		PQ219444-PQ219453	*Haemaphysalis hystricis* (OQ135148)	(Jinzhai county Anhui province in China)	99.84%-100.00%
		PQ219454-PQ219459	*Haemaphysalis longicornis* (MT465132)	Tick (Hanzhong, China)	99.68%-100.00%
	16S rRNA	PQ512796-PQ512797	*Ixodes ovatus* (NC_062061)	Hog-badger (Wuhan, China)	99.51%
		PQ512798-PQ512801	*Ixodes ovatus* (MH319616)	goat (Yunnan, China)	96.15%
		PQ512802-PQ512805	*Ixodes ovatus* (MH319598)	goat (Yunnan, China)	96.19%-96.65%
Rickettsia	16S rRNA	PQ203952-PQ203960	Uncultured *Rickettsia* sp. (ON016521)	*Haemaphysalis flava* (Jiangsu, China)	99.92%

		PQ203972	*Rickettsia japonica* (CP047359)	Human (Zhejiang, China)	100.00%
	*glt*A	PQ249177	*Rickettsia japonica* (CP047359)	Human (Zhejiang, China)	100.00%
		PQ249178-PQ249186	*Candidatus* Rickettsia principis (ON600642)	*Haemaphysalis flava* (Jiangsu, China)	100.00%
		PQ249175-PQ249176	Rickettsia raoultii（MK304547）	*Dermacentor reticulatus* (Russia)	100.00%
	groEL	PQ249190-PQ249198	*Candidatus* Rickettsia principis (PP791919)	Tick collected from vegetation (Rissua)	99.89%
		PQ249187-PQ249188	*Rickettsia raoultii* (CP010969)	Human (Inner Mongolia, China)	100.00%
		PQ249189	*Rickettsia japonica* (KX987373)	*Haemaphysalis hystricis* (Wuhan, China)	100.00%
Ehrlichia	16S rRNA	PQ205311	*Ehrlichia* sp. (KJ410252)	*Dermacentor nuttalli* (Xinjiang, China)	100.00%
		PQ205309	*Candidatus* Ehrlichia shimanensis (AB074459)	(USA)	100.00%
		PQ205310	Uncultured *Ehrlichia* sp. (FJ966350)	*Haemaphysalis japonica* (Russia)	100.00%
		PQ205312-PQ205314	*Ehrlichia chaffeensis* (NR_074500)	(USA)	99.53%,100.00%, 99.53%
		PQ205315	*Candidatus* Ehrlichia hainanensis (MT875365)	*Niviventer fulvescens* (Hainan, China)	100.00%
	groEL	PQ249204	*Ehrlichia chaffeensis* (CP000236)	(USA)	94.42%
		PQ249203	*Ehrlichia* sp. (KJ410295)	Tick (Xinjiang, China)	99.12%
		PQ249199	Uncultured *Ehrlichia* sp. (FJ966349)	*Haemaphysalis japonica* (Russia)	99.41%
		PQ249200-PQ249201	*Ehrlichia* sp. (OQ185236)	*Haemaphysalis hystricis* (Anhui, China)	100.00%
		PQ249202	*Candidatus* Ehrlichia zunyiensis (OM920706)	*Berylmys bowersi* (Guizhou, China)	100.00%
		PQ249205	*Candidatus* Ehrlichia shimanensis (AB074462)	Tick	94.71%
Anaplasma	16S rRNA	PQ206196-PQ206198	*Anaplasma bovis* (KY548389)	Raccoon dog (South Korea)	100.00%

		PQ206199-PQ206201	*Anaplasma capra* (LC432114)	*Hydropotes inermis argyropus* from Korean Water Deer (South Korea)	100.00%
	*glt*A	PQ249206-PQ249211	*Anaplasma bovis* (MH255921)	Goat (Shaanxi, China)	100.00%
		PQ249212-PQ249213	*Anaplasma capra* (OQ185246)	*Haemaphysalis longicornis* (Anhui, China)	100.00%
	groEL	PQ249217-PQ249219	*Anaplasma bovis* (MW122329)	Cattle blood (South Korea)	99.86%
		PQ249214-PQ249216	*Anaplasma bovis* (OQ702283)	*Haemaphysalis longicornis* (Hebei, China)	100.00%
		PQ249220-PQ249221	*Anaplasma capra* (OP974583)	Goat blood (China)	100.00%
Babesia	18S rRNA	PQ207054	*Babesia* sp. (AB935167)	(Japan)	100.00%
Theileria	18S rRNA	PQ207055	*Theileria capreoli* (JX134577)	White-lipped deer blood (China)	99.93%
		PQ207056-PQ207061	*Theileria luwenshuni* (MH208628)	*Rhipicephalus microplus* (Yunnan, China)	100.00%
		PQ207062	*Theileria orientalis* (MH208641)	*Rhipicephalus microplus* (Yunnan, China)	100.00%
Coxiella	IS*1111*	PQ249222-PQ249228	*Coxiella burnetii* (KX852471)	*Hyalomma asiaticum* (Xinjiang, China)	100.00%
		PQ249229-PQ249232	*Coxiella burnetii* (KX852467)	*Hyalomma asiaticum* (Xinjiang, China)	100.00%

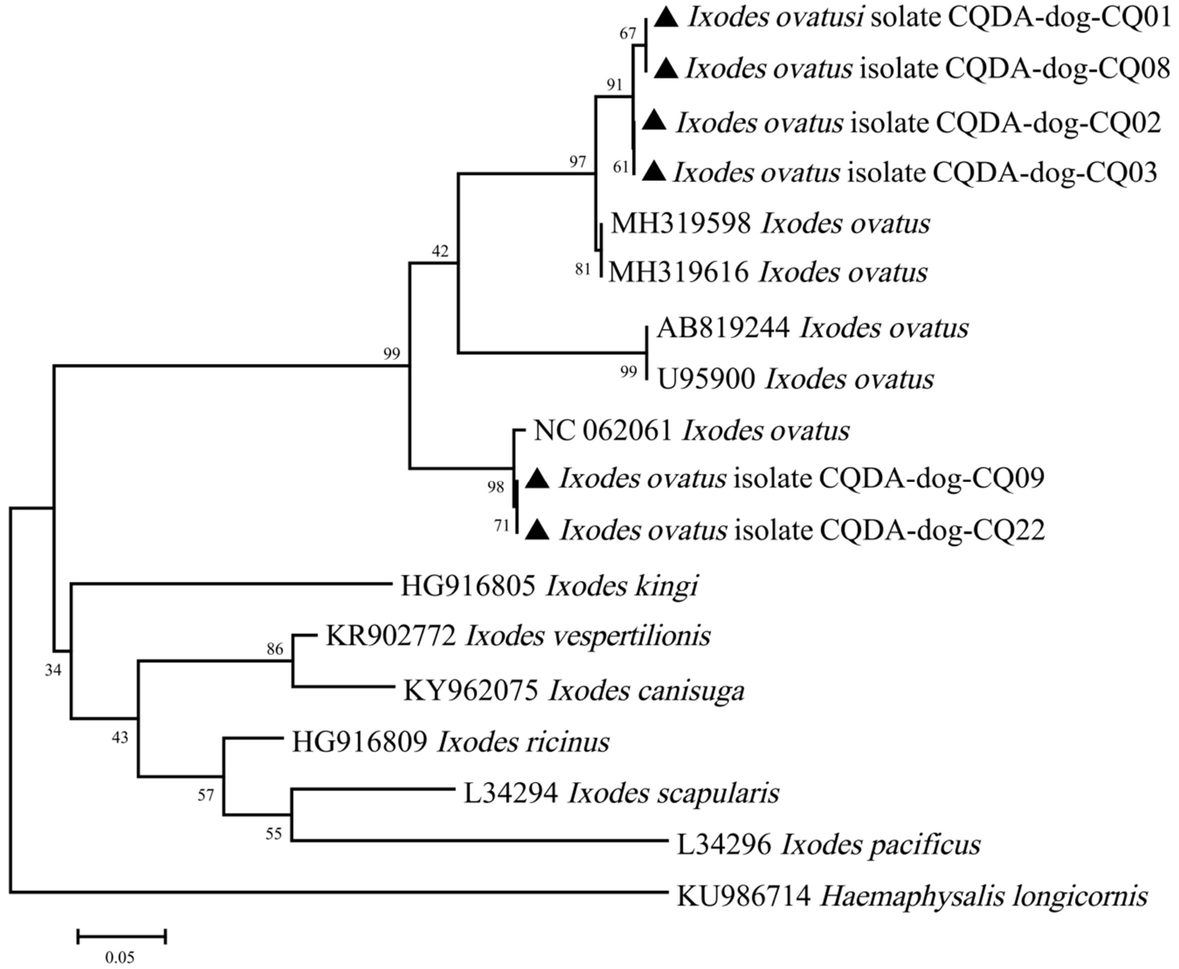

▲ Ixodes ovatusi solate CQDA-dog-CQ01
▲ Ixodes ovatus isolate CQDA-dog-CQ08
▲ Ixodes ovatus isolate CQDA-dog-CQ02
▲ Ixodes ovatus isolate CQDA-dog-CQ03
MH319598 Ixodes ovatus
MH319616 Ixodes ovatus
AB819244 Ixodes ovatus
U95900 Ixodes ovatus
NC 062061 Ixodes ovatus
▲ Ixodes ovatus isolate CQDA-dog-CQ09
▲ Ixodes ovatus isolate CQDA-dog-CQ22
HG916805 Ixodes kingi
KR902772 Ixodes vespertilionis
KY962075 Ixodes canisuga
HG916809 Ixodes ricinus
L34294 Ixodes scapularis
L34296 Ixodes pacificus
KU986714 Haemaphysalis longicornis
67
91
97
61
81
42
99
99
98
71
86
34
43
57
55
0.05

Figure S1. Phylogenetic analysis of ticks based on the nucleotide sequences of 16S rRNA. Sequences obtained in this study were marked with black triangles before their names.
